# Comparative Study of Physicochemical Properties of Alginate Composite Hydrogels Prepared by the Physical Blending and Electrostatic Assembly Methods

**DOI:** 10.3390/gels8120799

**Published:** 2022-12-05

**Authors:** Yanshi Wen, Xiuqiong Chen, Huiqiong Yan, Qiang Lin

**Affiliations:** 1 Key Laboratory of Water Pollution Treatment & Resource Reuse of Hainan Province, College of Chemistry and Chemical Engineering, Hainan Normal University, Haikou 571158, China; 2 Key Laboratory of Natural Polymer Functional Material of Haikou City, College of Chemistry and Chemical Engineering, Hainan Normal University, Haikou 571158, China; 3 Key Laboratory of Tropical Medicinal Resource Chemistry of Ministry of Education, College of Chemistry and Chemical Engineering, Hainan Normal University, Haikou 571158, China

**Keywords:** sodium alginate, physical blending, electrostatic assembly, composite hydrogels, tissue engineering

## Abstract

Alginate hydrogel commonly suffers from defects, such as weak mechanical properties, the shortage of long-term stability in physiological medium and the lack of mammalian cell adhesivity due to its strong hydrophilicity in biomedical application. For this reason, the homogeneous alginate hydrogels (Alg Gel) were successfully prepared by the D-glucono-δ-lactone/hydroxyapatite (HAP/GDL) cross-linking system, and then, the physical blending and alternating electrostatic assembly technology were proposed to fabricate alginate composite hydrogels (Alg-GT, Alg-CS-GT and ALG/GT-CS). The feasibility of the design methods was verified through the comparative analysis of their physicochemical properties and biological activity. In particular, the effects of physical blending and alternating electrostatic assembly technology on the pore structure, mechanical properties, swelling, degradation, cell adhesion and proliferation of composite hydrogels were also investigated. Experimental results showed that the formation of polyelectrolyte complexes by electrostatic assembly between biological macromolecules and the covalent cross-linking of EDC/NHS to GT improved the vulnerability of ion cross-linking, enhanced the mechanical properties and swelling stability of the composite hydrogels, and regulated their pore structure and in vitro biodegradability properties. Furthermore, MC3T3-E1 cells could exhibit good cell adhesion, cell viability and cell proliferation on the alginate composite hydrogels. Among them, Alg-CS-GT showed the best cell proliferation ability and differentiation effect due to its good cell adhesion. In view of the excellent physicochemical properties and biological activity of Alg-CS-GT, it exhibited great potential in biomedical application for tissue engineering.

## 1. Introduction

Many diseases and disasters can cause damage to some tissues and organs of the human body. In recent years, a variety of diseases caused by the damage or absence of tissues or organs have been the main causes of harm to human health. Although autologous transplantation and allotransplantation have achieved remarkable results in clinical practice, these methods are far from meeting the actual needs at the cost of damaging other healthy parts of the patient’s body and the health of the donor [1]. Recent studies have shown that three-dimensional (3D) porous composite hydrogel scaffolds constructed with natural biomacromolecules, such as alginate, have good biocompatibility and biodegradability, which can support and guide the adhesion, proliferation and differentiation of osteoblasts to maximize the recovery of damaged tissue function. Their emergence has solved the long-standing problem of treating tissue defects caused by severe trauma, tumor and deformity, and they are of great significance to the improvement of human life expectancy and quality of life [2,3].

In recent years, many natural polymers have been reported as raw materials for the preparation of hydrogel scaffolds, including hyaluronic acid, collagen and alginate [4]. Among these natural polymers, alginate, composed of 1,4-linked α-L-guluronic acid (G) and β-D mannuronic acid (M) units with random sequence distributions, was considered to be an ideal biomaterial for the fabrication of biomedical hydrogels due to its attractive merits, including source abundance, good biocompatibility, biodegradability, mild gelation conditions and encapsulation properties [5,6,7,8,9]. It can be cross-linked with divalent calcium ions under relatively mild pH and temperature conditions, and the formed alginate hydrogel with three-dimensional (3D) porous network structure that closely mimics the natural extracellular matrix (ECM) is capable of supporting crucial cellular activities, such as cell attachment, proliferation and differentiation [10,11]. It is reported that the human adipose-derived stem cells, human bone marrow mesenchymal stem cells and human umbilical vein endothelial cells could survive and form an extracellular matrix in alginate hydrogels, which promotes the exchange of nutrients and metabolites for cells [11,12].

However, alginate hydrogel still suffers from defects, such as weak mechanical properties, the shortage of long-term stability in physiological medium and the lack of mammalian cell adhesivity owing to its strong hydrophilicity, which seriously limit its application in the biomedical field [13,14,15]. To address these drawbacks, many attempts have been made to improve the physicochemical properties of alginate hydrogel. In particular, the physical blending approach and alternating electrostatic assembly between biological macromolecules for the construction of composite hydrogels may be the most effective approaches, as the composite materials not only eliminate the defects of the single alginate hydrogel but also endow them with many outstanding physicochemical properties and biological properties [9,16]. The physical blending method was applied to mix some bioactive nanomaterials into the alginate hydrogel matrix, so that the materials could be mixed with each other, achieving the synergistic effect. Tanpichai et al. [17] prepared cellulose nanocrystal/polyvinyl alcohol nanocomposite hydrogels, revealing their good mechanical properties and great potential in tissue engineering applications. Devin et al. [18] composed the porous composite matrix with hydroxyapatite and poly (lactic acid)/poly (hydroxyacetic acid) (50:50), and the results showed that the compressive elastic modulus of the composite material increased with the increase in hydroxyapatite composition. At the same time, the introduction of hydroxyapatite could improve the mechanical properties of poly (lactic acid)/poly (glycolic acid) and bone binding ability.

The ion cross-linking method of alginate is the main factor affecting its pore structure and morphology of the composite hydrogel. The commonly used exogenous cross-linking of calcium ions mainly relies on the penetration of calcium ions for cross-linking, which is susceptible to generating defects, such as uneven cross-linking and hydrogel deformation [19]. It has been reported that homogeneous hydrogels prepared with alginate through endogenous cross-linking can effectively improve the mechanical strength and mechanical integrity of composite hydrogels [20]. Endogenous cross-linking of alginate refers to homogeneous cross-linking caused by mixing insoluble salt of calcium and alginate solution evenly in advance and then lowering the pH of the solution by gluconolactone (GDL) to achieve the slow release of Ca^2+^ [8,9]. The commonly used insoluble salts of calcium are CaCO_3_, CaSO_4_ and Ca_3_(PO_4_)_2_, etc. During the cross-linking process, the release rate of Ca^2+^ from these inorganic salts under the action of acid hydrolysis of GDL is too slow, which can easily cause the gravity sedimentation of the inorganic salts, resulting in uneven cross-linking. In addition, the CO_2_ released from CaCO_3_ in the cross-linking process will make the pore size of the material too large, damaging its mechanical strength [14]. Therefore, it is an effective way to select an appropriate calcium salt to initiate the homogeneous cross-linking of alginate to enhance the structural and mechanical properties of the composite hydrogels.

In addition, as previously reported, the physical blending of ECM proteins, such as laminin, fibronectin and collagen, in the alginate matrix [21,22,23] or chemical coupling of Arg-Gly-Asp (RGD) peptide sequences can improve the cell adhesion of alginate [11]. However, it is generally difficult to control the physical mixing of proteins, and this often leads to non-specific interactions that trigger immune responses and protein hydrolysis [24], while chemically coupled RGD polypeptide sequences are based on carbonized diimide chemistry, which inevitably introduces organic solvents and reduces the biocompatibility of the material [19]. Therefore, choosing a reasonable method to improve the surface activity of alginate hydrogel is very important for the successful design and medical application of composite hydrogels. It is worth noting that hydroxyapatite (HAP), as the main inorganic component of bone tissue, is itself an insoluble calcium salt, which can be regarded as a basic complex salt composed of Ca_3_(PO_4_)_2_ and Ca(OH)_2_ [20]. Due to its good biocompatibility and bone conductivity, it is expected to be used as a cross-linking agent in the preparation of alginate homogeneous hydrogels. In addition, in nature, organisms are established by the precise assembly of biological macromolecules, which provides research ideas for the construction of tissue engineering hydrogel materials [25]. The use of alternating electrostatic assembly between biological macromolecules to modify material surfaces in a controlled manner is considered a novel and promising technology because it could realize layer-by-layer electrostatic self-assembly on the hydrogel’s surface, which improves its stability and biological activity in the physiological environment [26]. Alginate is a polyanionic electrolyte, which can form polyelectrolyte composite hydrogel through electrostatic interaction with polycationic electrolytes, such as chitosan, gelatin, poly (L-lysine) and poly (L-ornithine) [27,28]. As alginate and chitosan, and chitosan and B-type gelatin have opposite charges, they are able to form polyelectrolyte complexes. The Arg-Gly-Asp (RGD) peptide sequence contained in gelatin molecules is a specific protein for mutual recognition between cells and their extracellular matrix, which can regulate adhesion and physiological activity [22,23].

In the present work, in order to address the defects of alginate hydrogels, thereby improving their applicability in the biomedical field, homogeneous alginate hydrogels (Alg Gel) were prepared by the D-glucono-δ-lactone/hydroxyapatite (HAP/GDL) cross-linking system, and then, the positively charged chitosan (CS) and negatively charged type B gelatin (GT) were successively coated on the surface of alginate hydrogels by alternating electrostatic assembly technology. Since the GT molecule contains repeated RGD polypeptide sequences, it can help enhance the cell adhesion of composite hydrogels when it is covered on their outer surface. The feasibility of the design method was verified by elemental analysis and X-ray photoelectron spectroscopy. The effect of the HAP/GDL cross-linking system on the formation of alginate hydrogels was studied, and the effects of physical blending and alternating electrostatic assembly technology on the pore structure, mechanical properties, swelling, degradation and biological activity of composite hydrogels were investigated by comparative analysis. In particular, the cytotoxicity of alginate composite hydrogels, including cell adhesion, cell proliferation and cell differentiation, was also studied comparatively.

## 2. Results and Discussion

### 2.1. Fabrication of Homogeneous Alginate Composite Hydrogels

The homogeneous alginate composite hydrogels were fabricated via physical blending and alternating electrostatic assembly technology with the HAP/GDL complex as the endogenous cross-linking agent according to our previous method [29,30]. In the ion cross-linking of alginate, the cross-linking rate is the key factor to control the gel uniformity and strength. By uniformly dispersing Ca^2+^ and controlling the cross-linking rate, the homogeneity and mechanical strength of the gel material can be effectively promoted [20]. For this reason, insoluble calcium salt is usually mixed with alginate solution in advance, and then, the acidity of the solution is slowly reduced by adding acid ester, so that Ca^2+^ is gradually released, so as to achieve a controlled cross-linking of the hydrogel [31]. The morphology of the HAP nanoparticles used in this work is shown in Figure S1, and its particle size was about 65 nm. As a nanoparticle, HAP can be dispersed in SA solution more uniformly under mechanical action. When GDL is added to a homogeneous mixture of SA and HAP, alginate can form microgels with small amounts of Ca^2+^ released from HAP. The formation of the microgel increased the viscosity of the solution, which effectively prevented the gravitational deposition of HAP, thus producing a uniform hydrogel. In addition, HAP has good biocompatibility and osteoconductive properties. Therefore, the HAP/GDL complex can be used as a cross-linking agent to prepare ideal alginate homogeneous hydrogel materials.

**Figure 1:**
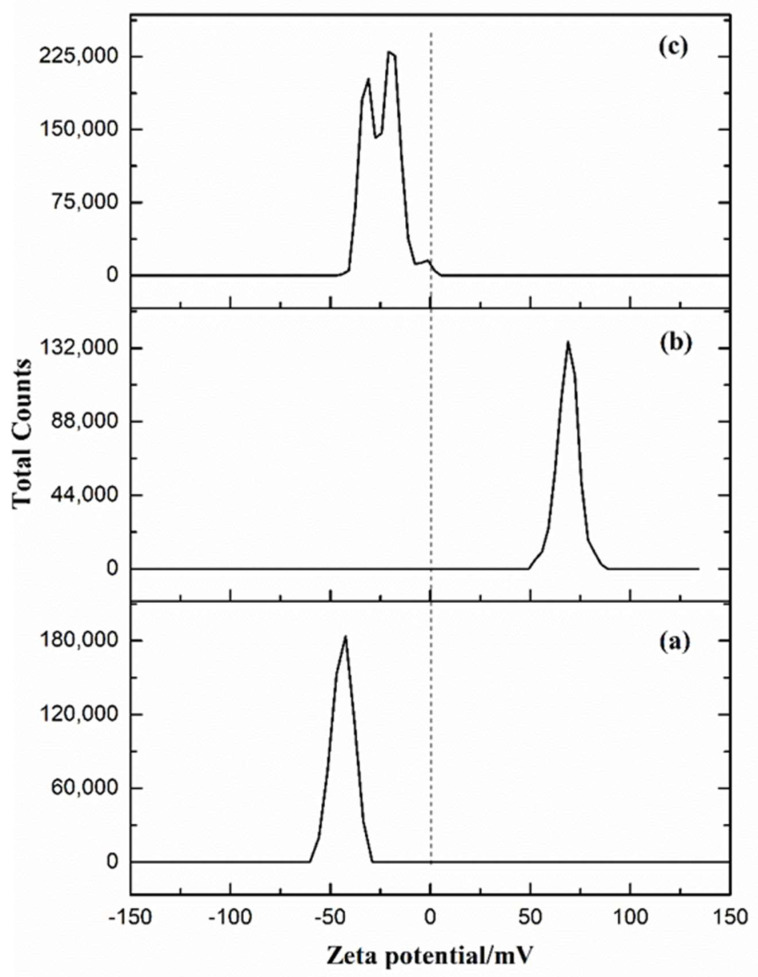
Zeta potentials of (**a**) SA, (**b**) CS and (**c**) Type B GT.

The electrostatic assembly of alginate hydrogels is based on the different charged properties of biomacromolecules. The zeta potentials of SA, CS and GT are shown in Figure 1, where they are able to form polyelectrolyte complexes due to the opposite charge between SA and CS, CS and type B GT [27,28]. Because GT has high swelling property in water, it usually needs to be cross-linked [32]. EDC/NHS is considered a class of mild cross-linking agents that do not disrupt the biological activity of collagen-like molecules [33]. The use of EDC/NHS as a cross-linking agent can not only improve the mechanical strength of the material but also improve its biological activity [34]. In this paper, the ion cross-linking induced by HAP-GDL, the electrostatic assembly between polyelectrolytes and the covalent cross-linking of EDCNHS are combined organically to create a fully simulated extracellular matrix environment for cell activity.

Due to the addition or deposition of other biomacromolecules, the composition of the composite hydrogels, such as C, H, O and N, would change accordingly, with the results shown in Table 1. The composition of each element is closely related to its molecular structure and functional group type. For example, the SA molecular chain mainly contains the carboxyl group and hydroxyl group, so its content of the O element was high. The CS molecular chain has a high content of the N element because of its amino group. However, GT is composed of amino acids, so its N element content is the highest. As can be seen from Table 1, the ALG Gel exhibited the highest O content due to its alginate characteristics. The content of the N element in Alg-GT increased significantly because of the coating of GT. Compared with Alg-GT, the decrease in N content in Alg-CS-GT was attributed to the CS overlay. However, they were lower than the N content of ALG/GT-CS prepared by the physical blending method. The results showed that the composition of the hydrogel was changed by electrostatic deposition between biomacromolecules under the electrostatic forces.

**Figure 2:**
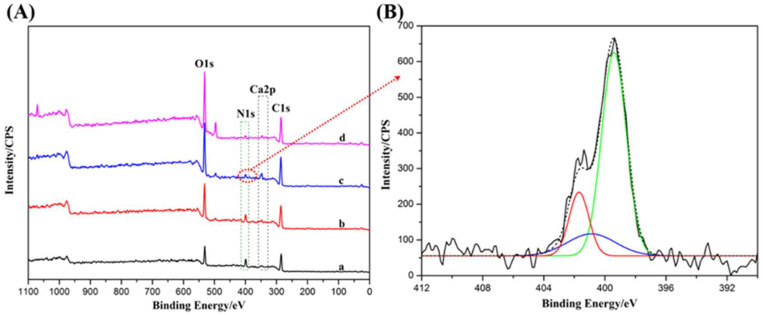
(**A**) XPS spectra of (a) Alg/GT-CS, (b) ALG-GT, (c) ALG-CS-GT and (d) Alg Gel, and (**B**) N1s spectra peak of ALG-CS-GT and their fitting curves.

Furthermore, the electrostatic assembly of biomacromolecules on the alginate hydrogel surface was further verified by XPS. XPS could effectively determine the element composition of the surface of the composite hydrogel, and the peaks of the elements were marked as shown in Figure 2A; the corresponding XPS element analysis results are shown in Table 2. The XPS elemental analysis results were similar to those in Table 1, further indicating that the electrostatic assembly of biomacromolecules took place on the surface of materials. However, Alg-CS-GT had the highest content of Ca, which was beneficial to the biomineralization of the composite hydrogel. In addition, by comparing the N1s peaks of ALG Gel, Alg-GT, Alg-CS-GT and ALG/GT-CS, it was found that the N1s peaks of Alg-CS-GT presented a three-peak structure. Three peaks located at 399.4 eV, 400.8 eV and 401.7 eV were obtained by curve fitting, which belonged to the binding of NH_2_, NHCO and NH_3_^+^, respectively [35]. The results showed that the amino groups of Alg-CS-GT existed in the composite hydrogel system in the form of physical, chemical coupling and ion binding simultaneously, which indicated that the electrostatic assembly between polyelectrolytes and EDC/NHS covalent cross-linking were feasible to modify the surface of alginate hydrogels.

### 2.2. Pore Structure and Mechanical Properties of the Composite Hydrogels

**Figure 3:**
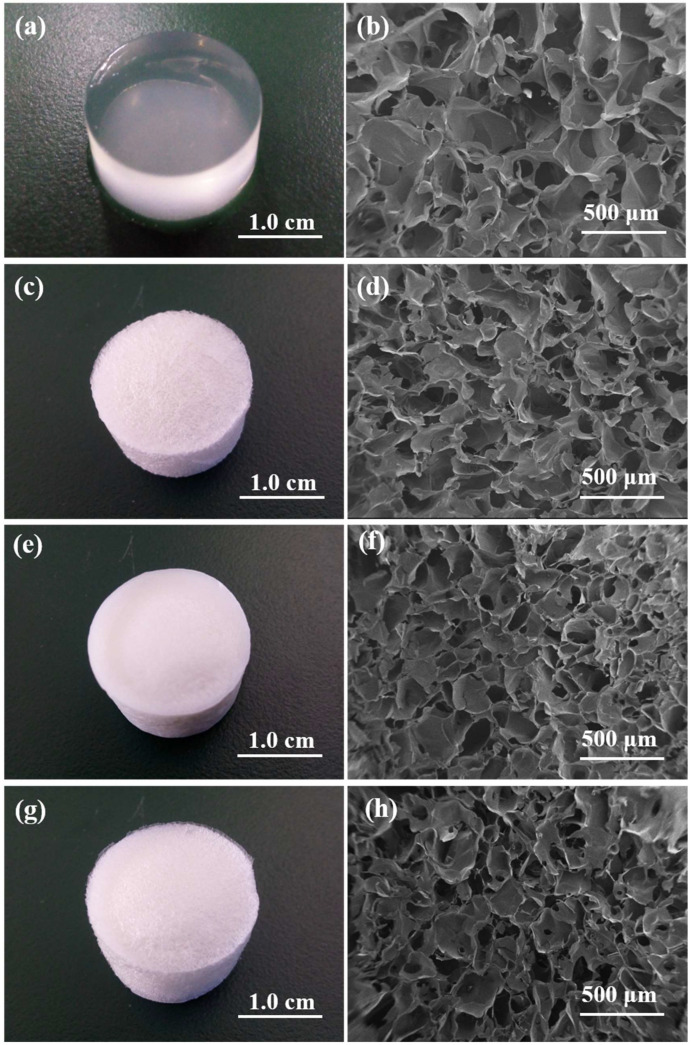
Photographs of (**a**) undried ALG Gel, (**c**) dried Alg-GT, (**e**) dried Alg-CS-GT and (**g**) dried ALG/GT-CS; SEM images of the cross-section of (**b**) ALG Gel, (**d**) Alg-GT, (**f**) Alg-CS-GT and (**h**) ALG/GT-CS.

The photographs of the alginate composite hydrogels and the SEM images of their pore structure are shown in Figure 3. All of the composite hydrogels exhibited good 3D morphology, which indicated that the preparation of homogeneous alginate hydrogels using HAP/GDL as the cross-linking agent was feasible [36]. Of note, Alg-GT, Alg-CS, Alg-CS-GT and ALG/GT-CS displayed similar pore structures, as shown in Figure 3b,d,f,h, implying that the electrostatic assembly of biomacromolecules did not destroy the porous structure of alginate composite hydrogels. The homogeneous hydrogels prepared with HAP/GDL as the cross-linking agent formed ice crystals uniformly in the hydrogels after freezing treatment, and the ice crystals were sublimated under negative pressure, leaving open porous structures in the hydrogels [37,38]. The porosities of the composite hydrogels measured by mercury porosimeter are shown in Table 3. The initial ALG Gel possessed high porosity (94.17%), and its pore size was in the range of 100~300 μm. As shown in Figure 3 and Table 3, after electrostatic assembly, the pore sizes of Alg-GT, Alg-CS-GT and ALG/GT-CS decreased significantly, and the porosity decreased due to the deposition of biomacromolecules. However, it still maintained a regular morphology of the pore structure. The porosity of ALG/GT-CS was the smallest because of the packing action of GT and the accumulation of CS.

**Figure 4:**
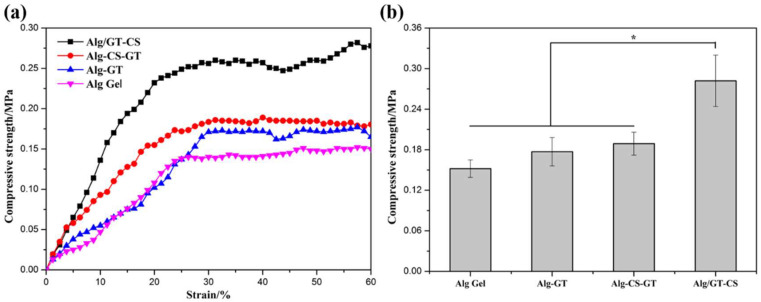
(**a**) Stress–strain curves and (**b**) compressive strength of ALG Gel, Alg-GT, Alg-CS-GT and ALG/GT-CS. * represents *p* < 0.05, indicating significant difference.

Alginate composite hydrogel as a medical material must have the appropriate mechanical strength to support the growth of cells and bear the burden of tissue [39,40]. Figure 4a shows the stress–strain curves of the alginate composite hydrogels. The highest linear point of each curve was the compressive strength of the composite hydrogel. Although the homogeneous ALG Gel was prepared by endogenous cross-linking, its mechanical properties were still only 0.132 MPA, as shown in Figure 4b. The compressive strength of Alg-GT and Alg-CS-GT was improved to some extent after electrostatic assembly of biomacromolecules. This was mainly due to the formation of polyelectrolyte complexes during electrostatic assembly and the covalent cross-linking of GT by EDC/NHS, which enhanced the vulnerability of the ionic cross-linking. At the same time, the mechanical properties of the composite hydrogels were improved because of the decrease in porosity due to the deposition and filling of biomacromolecules. In particular, when GT was mixed into the alginate matrix by the physical blending method, its mechanical properties were greatly improved due to its filling and intermolecular interaction [41]. Moreover, the improvement of the mechanical properties of Alg-GT/CS by this method was significantly higher than that by electrostatic assembly of biomacromolecules, which was related to the fact that the biomacromolecules involved in the assembly are less deposited in the material. Therefore, physical blending was still an effective method to improve the mechanical properties of the composite hydrogels.

### 2.3. Interactions between Components of Alginate Composite Hydrogels

The interactions between components directly affected the structure and properties of the composite hydrogel. In order to clarify the internal interactions between components, the functional group structure, crystal structure and thermal properties of the composite hydrogels were examined by FT-IR, XRD and TGA measurements, respectively.

**Figure 5:**
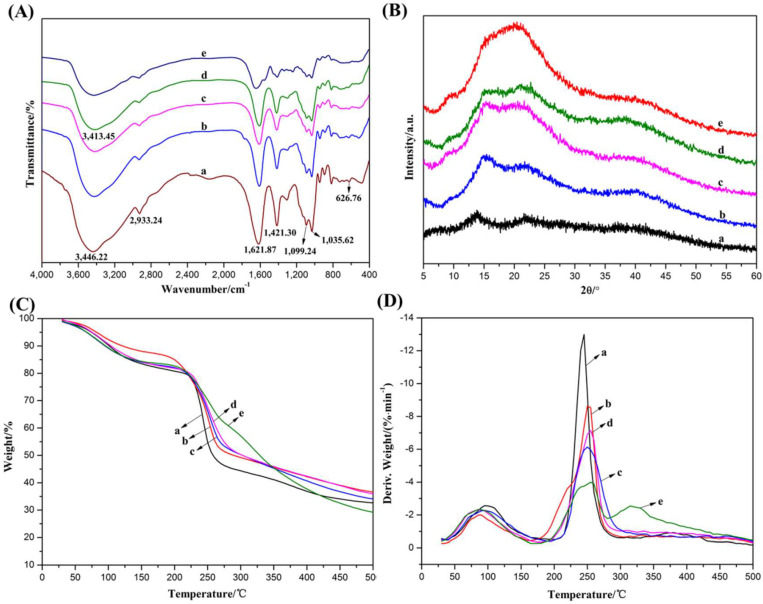
(**A**) FT-IR spectra, (**B**) X-ray diffractions, (**C**) TGA curves and (**D**) DTG curves of the composite hydrogels: (a) SA, (b) Alg Gel, (c) Alg-GT, (d) Alg-CS-GT and (e) Alg/GT-CS.

Figure 5A displays the FT-IR spectra of SA, ALG Gel, Alg-GT, Alg-CS-GT and ALG/GT-CS. Raw SA exhibited characteristic absorption peaks at 1621.87, 626.76 and 1421.30 cm^−1^, which were classified as asymmetric and symmetric stretching vibration absorption peaks of-COO^−^, respectively [42]. The small peak at 2933.24 cm^−1^ and the elastic vibration absorption peak at 1099.24 and 1035.62 cm^−1^ in the twin peaks belonged to C-H and C-O-C on the sugar skeleton, respectively [43]. The peak shape of SA did not change obviously when it was cross-linked by ion or when it was prepared by electrostatic assembly or physical blending with other biomolecules. However, in the range of 3000~4000 cm^−1^, the -OH stretching vibration absorption peak had a certain degree of deformation and red shift. In particular, Alg-CS-GT widened the absorption peak of OH stretching vibration at 3446.22 cm^−1^ and significantly red-shifted to 3413.45 cm^−1^. The above analysis results indicate that, in addition to electrostatic interactions, intermolecular hydrogen bonds between SA and various components are generated in alginate composite hydrogels [41].

The interaction between alginate and other components in alginate composite hydrogels was further verified by XRD analysis. As shown in Figure 5B, SA exhibited amorphous structural features. Because of the cross-linking of Ca^2+^, two broad peaks appeared at 2θ = 16.2° and 21.8°, which belonged to the hydrated crystal structure of calcium alginate [13,44]. However, the diffraction peak at 2θ = 16.2° decreased with the deposition and blending of GT. Especially on the diffraction curve of ALG/GT-CS, the diffraction peak at 2θ = 16.2° almost disappeared. These changes may be due to the formation of a polyelectrolyte complex by electrostatic interaction of SA with CS and GT or the formation of an intermolecular hydrogen bond that disrupted the hydrated crystalline structure of calcium alginate [43]. The results indicate that the hydrogen bond between SA and other components in the composite hydrogels may be formed.

Thermogravimetric analysis is the most effective method for characterizing the thermal stability of composite hydrogels, which is widely used to study interface interactions in composites [41]. Figure 5C presents the TGA curves of alginate composite hydrogels. It can be seen that at 500 °C, the weight loss rate of each sample was between 62% and 70%, which was attributed to the removal of physically adsorbed and water-bound composite hydrogels and the thermal cracking of polymer molecules themselves [45,46]. The main weightlessness process of composite hydrogels can be observed from their DTG curves. As shown in Figure 5D, all samples, except ALG/GT-CS, presented two major weightlessness stages, which ranged between 75~125 °C and 225~275 °C, respectively. The weightlessness at low temperature was mainly caused by the removal of physical adsorption water. Between 225 °C and 275 °C, however, the polymer began to undergo thermal cracking, where the molecular chain broke, and the polymer was gradually cracked into CO, CO_2_ and H_2_O, resulting in a rapid decrease in its weight [47,48,49]. However, different composite hydrogels exhibited different initial cracking temperatures, which reflected their different thermal stability. It was found that the initial pyrolysis temperature of the composite hydrogels was higher than that of SA, which indicated that the thermal stability of SA could be improved by ion cross-linking and the polyelectrolyte composite. Furthermore, Alg-CS-GT exhibited the highest thermal stability due to the fact that during electrostatic assembly, the close polyelectrolyte complex formed between SA and CS as well as CS and GT by electrostatic interaction or hydrogen bonding of intermolecular functional groups was helpful for improving the thermal stability of the composite hydrogels.

### 2.4. In Vitro Swelling and Biodegradation Analyses

**Figure 6:**
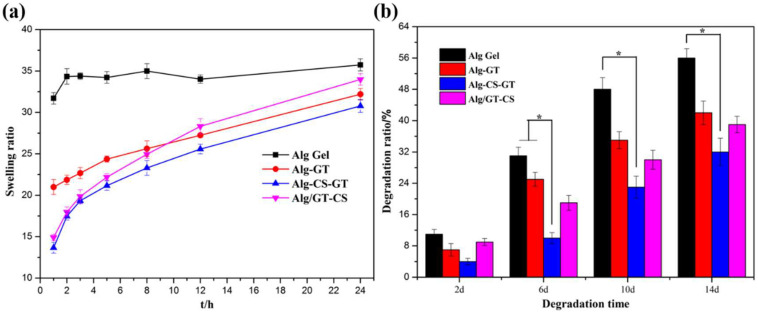
(**a**) Swelling behavior and (**b**) biodegradation rate of Alg Gel, Alg-GT, Alg-CS-GT and Alg/GT-CS. * represents *p* < 0.05, indicating significant difference.

The swelling property of composite hydrogels reflects their ability to adsorb media under physiological conditions [50]. Figure 6a shows the swelling curves of Alg Gel, Alg-GT, Alg-CS-GT and ALG/GT-CS in PBS at 37 °C. Because of SA’s strong hydrophilicity, Alg Gel revealed uncontrollable swelling in PBS, reaching up to more than 30 times its own weight after 1 h. For ALG Gel, excessive swelling could lead to the destruction of mechanical integrity, which was not conducive to the practical application of the materials [25,40]. Composite hydrogels prepared by electrostatic assembly and physical blending of biomacromolecules could significantly inhibit the swelling property of ALG Gel, enhancing its stability in saline solution. It was found that Alg-CS-GT had the best control of the swelling behavior, which was due to the formation of a dense polyelectrolyte complex during the electrostatic assembly process, which effectively restrained the swelling of composite hydrogels [30]. However, when the composite hydrogel was immersed in PBS for more than 12 h, ALG/GT-CS exhibited higher swelling due to the strong hydrophilicity of GT, which resulted in the continuous swelling of the material. As for Alg-GT, the outermost GT was cross-linked by EDC/NHS, which also improved its swelling stability in PBS.

The biodegradation of alginate composite hydrogels was performed in PBS containing 10,000 u/mL lysozyme because lysozyme was the most important enzyme in biodegradation, which could simulate the physiological environment [4]. Lysozyme performs degradation by interacting with the N-acetylglucosamine group on the CS chain [34]. As shown in Figure 6b, Alg Gel swelled dramatically in normal saline due to its uncontrollable swelling, and its calcium cross-linked structure was prone to exchange reaction with monovalent Na+ under the action of PO_4_^3+^, thus destroying its hydrogel structure. Therefore, Alg Gel was generally unstable under a physiological environment, and its degradation rate was fast. We found that compared with ALG Gel, Alg-CS-GT prepared by electrostatic assembly technology significantly reduced the biodegradability of the composite hydrogels. Alg-GT delayed the degradation of the composite hydrogels due to the deposition of GT and the covalent cross-linking of EDC/NHS. In addition, ALG/GT-CS prepared by the physical blending method had higher biodegradability than Alg-CS-GT because of its good hydrophilicity and the strong swelling property of the composite hydrogels, which increased its interaction area with lysozyme. The above results indicated that Alg-CS-GT prepared by the electrostatic assembly technology possessed a controllable degradation rate, which was of great significance for the matching of tissue regeneration [9].

### 2.5. Cytocompatibility

**Figure 7:**
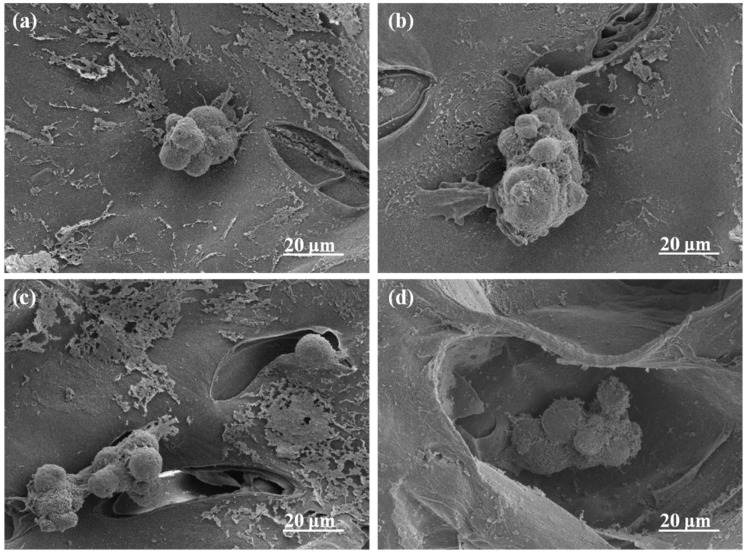
SEM images of MC3T3-E1 cells cultured on the composite hydrogels for 2 d: (**a**) Alg Gel, (**b**) Alg-GT, (**c**) Alg-CS-GT and (**d**) Alg/GT-CS.

After MC3T3-E1 cells were inoculated and cultured for 2 days, their growth morphology on the tissue culture plate was shown in Figure S2. MC3T3-E1 cells showed higher growth activity on the tissue culture plate, and the effect of proliferation was significant within 2 days. In addition, the adhesion and distribution of MC3T3-E1 cells on the alginate complex hydrogels is shown in Figure 7. Due to the lack of cell-specific binding sites of alginate, the adherent cells on ALG Gel were few in number, poorly dispersed and mainly concentrated in the vicinity of pores. In contrast, Alg-GT and Alg-CS-GT showed good cell adhesion and dispersion, and cells were distributed in the pores and pore wall of the material. This was mainly ascribed to the RGD polypeptide contained in the outermost GT of the composite hydrogel, which could effectively improve the cell adhesion of the material [51,52]. Moreover, the use of EDC/NHS as a cross-linking agent not only improved the mechanical strength of the material but also enhanced its biological activity [7]. However, ALG/GT-CS was more distributed on the inner side of the pore wall because its GT was mechanically mixed in the hydrogel material, and the cell adhesion effect was not ideal. The results implied that the electrostatic assembly of biomacromolecules could effectively improve the surface activity of composite hydrogels, thereby enhancing their cell adhesion.

**Figure 8:**
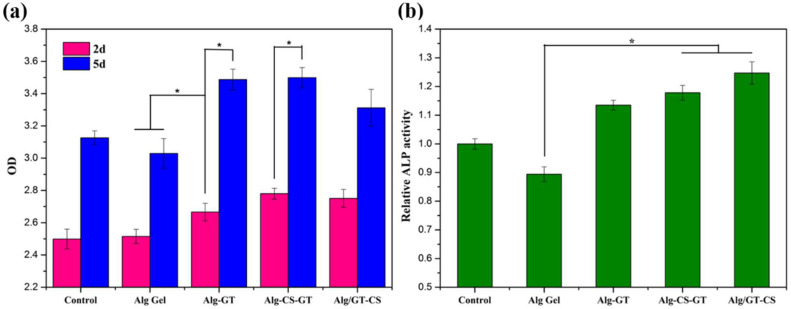
(**a**) Cell proliferation viability of MC3T3-E1 cells cultured on the Alg Gel, Alg-GT, Alg-CS-GT and Alg/GT-CS for 2 d and 5 d; (**b**) Cell differentiation of MC3T3-E1 cells on the Alg Gel, Alg-GT, Alg-CS-GT and Alg/GT-CS for 7 d. * represents *p* < 0.05, indicating highly significant difference.

After cells were cultured on alginate composite hydrogels for 2 and 5 days, respectively, there were some differences in proliferation due to different composite hydrogel materials, as shown in Figure 8a. Among them, the proliferation effect of cells on ALG-GT, ALG-CS-GT and Alg/GT-CS was significant, indicating that cells could show good cell viability and proliferation ability in such composite hydrogels. Alg-GT and Alg-CS-GT showed the best cell proliferation ability due to their good cell adhesion. Moreover, the proliferation effect of the composite hydrogel was significantly better than that of Alg Gel and the blank control group. A possible reason is that there was limited space for cells to grow in the tissue culture plate, which limited their growth [4]. As shown in Figure S2, the cells almost filled the whole tissue culture plate within 2 days. However, the OD value of alginate composite hydrogels was higher than that of the control because of their good 3D morphology and porous structure, which could provide space for the three-dimensional proliferation of cells [37]. The low proliferative activity of ALG Gel may be related to its lack of cell-specific binding sites [30]. However, during the preparation of ALG/GT-CS, GT was mechanically embedded in the alginate hydrogel, which resulted in lower proliferation activity of cells than Alg-GT and Alg-CS-GT. Therefore, GT in the composite hydrogel material, especially by electrostatic assembly on the composite hydrogels’ surface, had a great promotion effect on cell proliferation.

Since ALP is an important biological indicator of differentiation of early osteoblasts [53], the differentiation of cells on different composite hydrogels can be assessed by the relative ALP activity of cells. As shown in Figure 8b, after 7 days of culture, the ALP activity on alginate composite hydrogel was increased to a certain extent compared with that of the control group, and the ALP activity on ALG-CS-GT and Alg/GT-CS was significantly higher than that of the control group and Alg Gel. Among them, Alg Gel revealed poor cell adhesion, and its ALP activity was low. However, Alg/GT-CS showed the highest ALP activity during cell differentiation. This result may be related to the CS contained in the composite hydrogel because CS has a similar structure to glucosamine, the main active component of ECM, which can promote cell proliferation and differentiation [54,55]. Therefore, CS played an important role in cell proliferation and differentiation in composite hydrogels.

## 3. Conclusions

In summary, alginate homogeneous hydrogels (Alg Gel) were successfully prepared by the HAP/GDL cross-linking system, and then, the physical blending and alternating electrostatic assembly technology were applied to fabricate alginate composite hydrogels (Alg-GT, Alg-CS-GT and ALG/GT-CS). The feasibility of the design methods was verified through the comparative analysis of their physicochemical properties and biological activity. Meanwhile, the effects of physical blending and alternating electrostatic assembly technology on the pore structure, mechanical properties, swelling, degradation, cell adhesion and proliferation of composite hydrogels were also investigated. The results of elemental analysis and XPS indicated that the surface modification of the composite hydrogels was feasible by the electrostatic assembly of biomacromolecules. The alginate composite hydrogels prepared by the endogenous cross-linking of the HAP/GDL system and the electrostatic assembly of biomacromolecules displayed good 3D morphology and uniform pore structure. The formation of polyelectrolyte complexes during electrostatic assembly and the covalent cross-linking of EDC/NHS to GT improved the vulnerability of ion cross-linking and enhanced the mechanical properties and swelling stability of the composite hydrogels. The in vitro cell compatibility test showed that the RGD polypeptide contained in GT coated on the outermost layer of the material by electrostatic assembly technology could effectively improve the cell adhesion of the composite hydrogels, indicating that electrostatic assembly between biological macromolecules could effectively enhance the surface activity of the composite hydrogels. Moreover, MC3T3-E1 cells could exhibit good cell viability and proliferation on alginate composite hydrogels. Compared with other composite hydrogels, Alg-CS-GT showed the best cell proliferation ability and differentiation effect due to its good cell adhesion. The above analysis results suggested that Alg-CS-GT could be used as an ideal scaffold material in tissue engineering due to its good 3D morphology, uniform pore structure, suitable mechanical properties, controllable swelling and biodegradation properties, and excellent cytocompatibility.

## 4. Materials and Methods

### 4.1. Materials and Reagents

Sodium alginate (SA, M_W_ = 1,106,340, biological grade), hydroxyapatite (HAP, ≥97 wt%), positively charged chitosan (CS, M_W_ = 860,260, biological grade), D-glucono-δ-lactone (GDL), 1-ethyl-(3-dimethylaminopropyl) carbodiimide hydrochloride (EDC, ≥98 wt%), type A or type B gelatin (GT, biological grade) and N-hydroxysuccinimide (NHS, ≥97 wt%) were purchased from Aladdin Biochemical Technology Co., Ltd. (Shanghai, China). Mouse osteoblastic MC3T3-E1 cells were obtained from the cell bank of the Chinese Academy of Sciences, (Shanghai, China). MEM-α medium, trypsin (0.25% trypsin-EDTA), streptomycin, fetal bovine serum, penicillin and lysozyme (≥20,000 U/mg) were purchased from Amresco Co., Ltd. (Cleveland, OH, USA). Cell Counting Kit-8 (CCK-8) and alkaline phosphatase (ALP) assay kit were bought from Gibco (Thermo Fisher Scientific, Waltham, MA, USA).

### 4.2. Fabrication of the Composite Hydrogels

The fabrication of composite hydrogels was achieved by the physical blending method using the HAP/GDL complex as the endogenous cross-linking agent, followed by alternate electrostatic assembly of CS and GT, according to our previous report [29,30]. Briefly, 0.19 g of HAP powder was fully dispersed in 100 mL of 2% (*w*/*v*) SA aqueous solution under ultrasonic stirring. Then, the ion cross-linking of SA was initiated by the addition of 0.65 g of GDL under magnetic stirring. After stirring at high speed for 3 min, the mixture was quickly transferred into a 12-well tissue culture plate and physically cross-linked at 4 °C for a period of 24 h. Subsequently, the formed hydrogels were repeatedly washed with deionized water and then lyophilized to obtain the dried alginate hydrogel (Alg Gel). To further improve their cell adhesion and stability, the alternate electrostatic assembly of CS and GT was performed by soaking them in 1% (*w*/*v*) CS solution for a period of 30 min, followed by freeze-drying, and then soaking them in 1% (*w*/*v*) type B GT solution for a period of 30 min, followed by freeze-drying. Afterward, the obtained composite hydrogel was immersed into EDC/NHS (10 mM/10 mM) blend solutions in the presence of 0.01 mol/L CaCl_2_ for 12 h. Finally, it was washed with deionized water and lyophilized to obtain the dried Alg-CS-GT. For comparison, Alg Gel was directly immersed in 1% (*w*/*v*) type A GT solution based on the above method, and then, it was covalently cross-linked by EDC/NHS blend solutions to fabricate the ALG-GT composite hydrogel. Meanwhile, 2.0 g of type B GT was fully dissolved in 100 mL of 2% (*w*/*v*) SA aqueous solution under ultrasonic stirring, and then, the blend solutions were endogenously cross-linked by the HAP–GDL complex and coated with the CS solution to obtain the Alg/GT-CS composite hydrogel.

### 4.3. Characterization of the Composite Hydrogels

The Zeta potentials of SA, CS and GT were measured by a Zeta potential analyzer (Nano-ZS90, Malvern, UK). Each sample was prepared at a concentration of 0.1 wt%, and the test was carried out at 25 °C. An elemental analyzer (Vario EL Cube, Elementar, Frankfurt, Germany) was used to analyze the C, H, O and N elements of the prepared composite hydrogels. An X-ray photoelectron spectroscopy analyzer (Axis Ultra XPS, Kratos, Manchester, England, UK) was used to analyze and detect the elemental composition of the surface of each composite hydrogel. The porous morphology and porosity of the composite hydrogels were measured by a JSM-7100F scanning electron microscope (JEOL, Tokyo, Japan) and an AutoPore IV 9500 Mercury porosimeter (Micromeritics Instrument Co., Ltd., Shanghai, China). The functional group analysis of the composite hydrogels was conducted using a Nicolet-6700 Fourier transform infrared spectrophotometer (Thermo Scientific, Waltham, MA, USA). Before measurement, a small amount of dried sample and KBr were mixed, ground and compressed into discs for the test. XRD analysis of the sample powders was performed using a Rigaku UItima-IV X-ray diffraction (Nishiku Corporation, Tokyo, Japan). The Cu-Kα radiation was used as the X-ray source, the test voltage and current were 40 kV and 40 mA, respectively, and the XRD patterns of the samples were collected at room temperature at a scanning speed of 1.8 °/min over a 2θ range of 5~65°. Furthermore, a TA Q600 thermogravimetric analyzer (TA Instrument, New Castle, DE, USA) was used to analyze the thermal stability of various samples under N_2_ atmosphere at a heating rate of 10 °C/min with the test range of 25~500 °C. The mechanical property of samples was determined with a WDW-1 computer-controlled electronic universal tensile testing machine (Jinan Yinuo Century Testing Instrument Co., Ltd., Jinan, China). The freeze-dried sample was placed under the compression disc, and it was longitudinally compressed perpendicular to the surface of the sample. When the sample was compressed to a strain greater than 60%, it was stopped, and the compression speed was 5 mm/min. The highest linear point on the recorded stress–strain curve was the compressive strength of the sample. Each sample was measured five times in parallel and then averaged.

### 4.4. Swelling and In Vitro Biodegradation Studies

The swelling behavior of the composite hydrogels was measured in PBS buffer at 37 °C. The test samples were immersed in PBS solution (pH = 7.4) and then they were placed in an incubator at 37 °C for a certain time. After the specific time interval, they were gradually removed and a filter paper was used to gently wipe the residual PBS solution on their surface. The mass of samples before and after swelling was denoted as *W_dry_* and *W_wet_*, respectively. Each group was repeated 3 times to take the average value, and the swelling rate (SR) of the sample at each time interval was calculated according to the following Equation (1).

(1) $$\mathrm{SR}=\frac{W_{wet}-W_{dry}}{W_{dry}}$$

The biodegradation study of the composite hydrogels was conducted in PBS solution containing 10,000 U/mL lysozyme at 37 °C for a period of 2 d, 6 d, 10 d and 14 d. The dry samples weighted as *W_dry_* were immersed in the above simulated physiological conditions. At the set time intervals, the samples were gradually removed from the solution, washed with ultrapure water, and freeze-dried to weight their masses as *W_degradation_*. Three parallel experiments were performed for each group and the mean value was taken. The biodegradation ratio (BR) of the sample was calculated based on the mass change of the composite hydrogels after the biodegradation with the following Equation (2).

(2) $$\mathrm{BR}=\frac{W_{dry}-W_{degradation}}{W_{dry}}\times 100\%$$

### 4.5. Cytocompatibility of the Composite Hydrogels

Mouse osteoblast MC3T3-E1 cells, which can simulate the growth process of osteoblasts, were selected as the model cells and cultured in the medium composed of 90% DMEM, 10% fetal bovine serum, 100 U/mL penicillin and 100 μg/mL streptomycin to test the cytocompatibility of the composite hydrogel. The composite hydrogels with a diameter of 14 mm and a height of 3 mm were prepared and irradiated with cobalt 60 at an intensity of 8 kGy and then placed in the 24-well tissue culture plates. Subsequently, MC3T3-E1 cells were seeded on the composite hydrogel at a density of 5 × 10^4^ per well, while the same cells were seeded on the material-free tissue culture plate as the blank control. Meanwhile, the culture medium was then supplemented to maintain the total amount of medium up to 500 μL per well. Additionally, the cells were cultured in an incubator containing 5% CO_2_, 95% air and 100% relative humidity at 37 °C, and the medium was changed every 2 d.

After incubation for 2 d, the adhesion and spread of the cells on the nanocomposite hydrogels were examined by SEM observation after they were fixed by chemical cross-linking with glutaraldehyde followed by dehydration in graded ethanol series and freeze-drying. Afterward, the Cell counting Kit-8 (CCK-8) assay was used to detect the proliferation activity of the cells on the composite hydrogel. After 2 and 7 days of incubation, 50 μL of CCK-8 reagent was added to 500 μL of the medium in each well of 24-well culture plates, and the culture was incubated for 4 h at 37 °C in an incubator. Finally, 100 μL of solution per well was transferred to a 96-well plate. The OD value was measured by a Bio-rad X-mark microplate reader (Bio-Rad, Hercules, CA, USA) at a wavelength of 450 nm.

The osteogenic differentiation activity of the cells on the nanocomposite hydrogels was examined by the alkaline phosphatase (ALP) kit assay. The culture medium was supplemented with 0.2 μmol/L dexamethasone and 8.0 mmol/L β-glycerophosphate to promote cell differentiation. After incubation for 7 d, the cells were washed with PBS and disrupted in a 0.2% (*w*/*w*) Triton X-100 ice bath for 30 min, followed by centrifugation at 12,000 r/min for 5 min at 4 °C. Furthermore, 50 μL of the centrifugation supernatant was placed on a 96-well plate; then, 50 μL of the ALP staining reagent was added and fully mixed. After incubation at 37 °C for 30 min, the OD value was determined by X-mark microplate reader at 405 nm wavelength, which could be used as an indicator of the relative activity of ALP.

## Figures and Tables

**Table 1:** Elemental analysis results of Alg Gel, ALG-GT, ALG-CS-GT and Alg/GT-CS.

Sample	%C	%H	%O	%N
Alg Gel	30.67	4.91	47.31	0.43
Alg-GT	34.29	5.47	42.39	4.33
Alg-CS-GT	31.62	5.55	46.87	2.13
Alg/GT-CS	38.51	6.27	40.63	8.24

**Table 2:** XPS elemental analysis results for Alg Gel, ALG-GT, ALG-CS-GT and Alg/GT-CS.

Sample	%C	%N	%O	%Ca
Alg Gel	54.25	1.93	43.23	0.60
Alg-GT	58.98	9.51	30.56	0.95
Alg-CS-GT	57.68	3.79	36.77	1.76
Alg/GT-CS	63.46	12.49	23.06	0.70

**Table 3:** Porosity of ALG Gel, Alg-GT, Alg-CS-GT and ALG/GT-CS.

Samples	Porosity
ALG Gel	94.17%
Alg-GT	91.40%
Alg-CS-GT	90.35%
ALG/GT-CS	85.24%

## Data Availability

The data presented in this study are available on request from the corresponding author.

## Supplementary Materials

The following supporting information can be downloaded at: https://www.mdpi.com/article/10.3390/gels8120799/s1, Figure S1: TEM images of HAP nanoparticles at different magnifications; Figure S2: Optical micrographs of (a,b) MC3T3-E1 cells cultured on tissue culture plates at different magnifications.
