# Supplementary material for: Comparative Study of Physicochemical Properties of Alginate Composite Hydrogels Prepared by the Physical Blending and Electrostatic Assembly Methods
Source: Gels. 2022 Dec 5;8(12):799. doi: 10.3390/gels8120799 (PMC9777933; doi:10.3390/gels8120799)
Supplement: Supplementary file 1 [file gels-08-00799-s001.zip › gels-2028905-supplementary.pdf]

Support Information

Comparative study of physicochemical properties for alginate composite hydrogels prepared by the physical blending and electrostatic assembly methods

Yanshi Wen [a,b,c], Xiuqiong Chen [a,b,c], Huiqiong Yan [a,b,c]*, Qiang Lin [a,b,c]

[a] Key Laboratory of Water Pollution Treatment & Resource Reuse of Hainan province, College of chemistry and chemical engineering, Hainan Normal University, Haikou 571158, China

[b] Key Laboratory of Natural Polymer Functional Material of Haikou City, College of chemistry and chemical engineering, Hainan Normal University, Haikou 571158, China

[c] Key Laboratory of Tropical Medicinal Resource Chemistry of Ministry of Education, College of Chemistry and Chemical Engineering, Hainan Normal University, Haikou 571158, China

*Correspondence: Tel.: yanhqedu@163.com; +86 0898 65884995

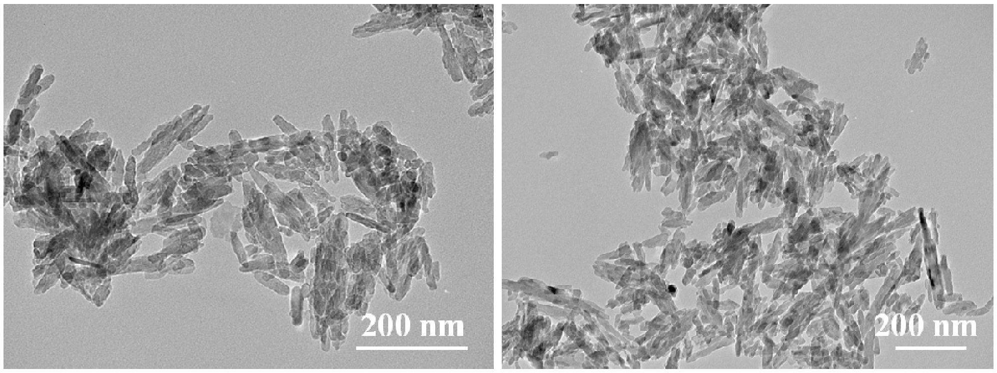

Figure S1. TEM images of HAP nanoparticles at different magnifications.

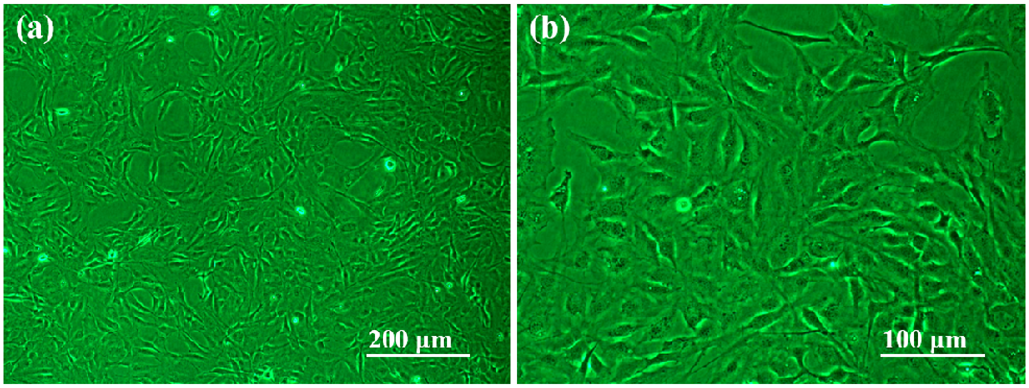

Figure S2. Optical micrographs of (a, b) MC3T3-E1 cells cultured on tissue culture plates at different magnifications.
